# Indications for accurate and appropriate use of personal protective equipment for healthcare professionals. A systematic review

**DOI:** 10.1590/1516-3180.2021.0128.R1.18052021

**Published:** 2021-12-17

**Authors:** Maria Stella Peccin, Márcio Luís Duarte, Aline Mizusaki Imoto, Mônica Taminato, Humberto Saconato, Maria Eduarda Puga, Eduardo Signorini Bicas Franco, Erika Barbosa Camargo, Leila Bernarda Donato Gottems, Álvaro Nagib Atallah

**Affiliations:** I PT, PhD. Associate Professor, Department of Human Movement Sciences, and Advisor, Evidence-Based Health Program, Universidade Federal de São Paulo (UNIFESP), São Paulo (SP), Brazil.; II MD, MSc. Musculoskeletal Radiologist, WEBIMAGEM Telerradiologia, São Paulo (SP), Brazil; and Doctoral Student in Evidence-Based Health Program, Universidade Federal de São Paulo (UNIFESP), São Paulo (SP), Brazil.; III PT, PhD. Physiotherapist and Professor, Professional and Academic Master’s Program, Laboratory for Evidence-Based Healthcare, Escola Superior de Ciências da Saúde, Hospital das Forças Armadas, Brasília (DF), Brazil.; IV PhD. Nurse and Associate Professor, Escola Paulista de Enfermagem, Universidade Federal de São Paulo, São Paulo (SP), Brazil.; V MD, PhD. Adjunct Professor, Discipline of Emergency and Evidence-Based Medicine, Universidade Federal de São Paulo (UNIFESP), and Researcher, Cochrane Brazil, São Paulo (SP), Brazil.; VI MSc, PhD. Librarian, Evidence-Based Health Program, Universidade Federal de São Paulo (UNIFESP), São Paulo (SP), Brazil.; VII PT, MSc. Doctoral Student in Evidence-Based Health Program, Universidade Federal de São Paulo (UNIFESP), São Paulo (SP), Brazil.; VIII MD, PhD. Professor, Postgraduate Program on Public Health Policies, Escola de Governo em Saúde (EGS), Fundação Oswaldo Cruz (Fiocruz), Brasília (DF), Brazil.; IX PhD. Professor, Professional and Academic Master’s Program, Laboratory for Evidence-Based Healthcare, Escola Superior em Ciências da Saúde (ESCS), Brasília (DF), Brazil.; X MD, PhD. Head of Evidence-Based Health Department, Universidade Federal de São Paulo (UNIFESP), São Paulo (SP), Brazil.

**Keywords:** COVID-19 [supplementary concept], Coronavirus infections, Disinfection, Respiratory, SARS-CoV-2, Mask disinfection, Face shield disinfection

## Abstract

**BACKGROUND:**

The speed of the spread of coronavirus disease 2019 (COVID-19) has put enormous pressure on hospitals and other healthcare facilities. This, together with blockages in several countries, has hindered the availability and accessibility of the necessary personal protective equipment (PPE).

**OBJECTIVE:**

To identify, systematically evaluate and summarize the available scientific evidence on the efficacy, safety, safe use and reuse of PPE for healthcare professionals, for preventing severe acute respiratory syndrome coronavirus 2 (SARS-CoV-2) infection.

**DESIGN AND SETTING:**

Systematic review of studies analyzing products for disinfecting and enabling reuse of PPE for coronavirus within the evidence-based health program of a federal university in São Paulo (SP), Brazil.

**METHODS:**

A systematic search of the relevant literature was conducted in the PubMed, EMBASE, Cochrane Library, CINAHL, SCOPUS, Web of Science and LILACS databases, for articles published up to November 30, 2020.

**RESULTS:**

Ten studies were selected. These analyzed the use of N95, surgical and cotton masks, face shields, flexible enclosures with plastic covers or polycarbonate intubation boxes and plastic curtains; and also PPE disinfection using several substances.

**CONCLUSION:**

Combined use of a face shield with a N95 mask proved to be superior to other associations for protecting healthcare workers. Some products are useful for disinfecting PPE, such as 70% ethanol, 0.1% sodium hypochlorite and a mixture of quaternary ammonium and H_2_O_2_, and hydrogen peroxide. Ultraviolet light and dry heat at 70 °C can be used to decontaminate N95 masks.

**REGISTRATION NUMBER:**

DOI: 10.17605/OSF.IO/4V5FD at the OPENSCIENCE Framework.

## INTRODUCTION

The pandemic caused by coronavirus disease 2019 (COVID-19) is severely affecting healthcare systems worldwide, including the care of several chronic diseases, such as cancer.^1^^,^^2^^,^^3^ Severe acute respiratory syndrome coronavirus 2 (SARS-CoV-2), the agent that causes COVID-19, is a respiratory virus transmitted through droplets and by contact. It can be disseminated through aerosolization, swab collection, intubation, aspiration, noninvasive ventilation, high-flow nasal cannulas and bag-mask ventilation.^4^^,^^5^ Prevention and control measures for the new coronavirus need to include hand hygiene, disinfection of surfaces (notably those frequently touched), avoidance of touching the face, respiratory manners (covering the mouth while coughing) and use of masks.^1^^,^^4^^,^^6^

The speed of the spread of COVID-19 has put enormous pressure on hospitals and other healthcare facilities.^7^ This, together with blockages in several countries, has hindered availability and accessibility regarding the necessary personal protective equipment (PPE).^7^ The Centers for Disease Control and Prevention (CDC) of the United States recommends the use of gloves, aprons, respiratory protection (e.g. disposable N95 respirators) and eye protection (e.g. goggles or face shields), without the use of shoe protectors (props).^8^ According to a meta-analysis by Li et al.,^9^ use of face masks decreased the risk of COVID-19 infection by 70%, for healthcare workers.

PPE in healthcare is generally considered to be part of what is called transmission-based precautions.^10^ Standard precautions or universal precautions are based on the principle that all blood, body fluids, secretions, excretions other than sweat, non-intact skin and mucous membranes can contain transmissible agents for infectious diseases.^10^ Depending on the expected exposure, hand hygiene and the use of PPE, such as gloves, aprons, masks, caps or eye protection (i.e. goggles or face protection) should be implemented.^10^ According to the World Health Organization, more than 59 million people work in the healthcare sector worldwide.^10^ These healthcare professionals are at risk of developing life-threatening infectious diseases through contact with patients’ blood or body fluids, such as mucus, vomit or exhaled drops.^10^

Sprays and splashes of fluids containing infectious microorganisms represent an occupational risk for healthcare professionals.^11^ The droplets of these fluids can be inhaled, come into contact with damaged skin or be deposited on the mucous membranes of the mouth, nose or eyes.^11^ Once in these structures, pathogens can infect workers and cause disease.^11^ It should be considered that small aerosol droplets from a patient with a cough can remain in the air and spread throughout a room, and can easily be inhaled by a healthcare professional.^11^^,^^12^

The risk of infection and its consequences are variable but are well recognized as an occupational risk.^10^ However, in epidemics, the risk of infection is higher because of the higher infection rate among healthcare professionals than among the general population.^5^^,^^10^ The variable clinical spectrum of COVID-19 needs to be considered: given that the majority of cases are asymptomatic or oligosymptomatic,^2^ infection can be passed from an asymptomatic healthcare professional to a patient with any other disease, or it can even be passed among the patients themselves.^10^ Furthermore, if healthcare professionals become infected, this decreases the capacity of the healthcare system to provide care, particularly at times of epidemic, when it is overburdened.^10^

This situation was previously experienced in 2002 and 2003, during the epidemic of the severe acute respiratory syndrome (SARS), in which 20% of all patients were healthcare professionals and about 10% lost their lives.^10^^,^^13^^,^^14^ The scarcity of PPE and its ineffective implementation were the main reasons behind the high number of healthcare professionals who became infected at the beginning of the COVID-19 pandemic.^7^ In March 2020, Remuzzi et al. reported that a fifth of healthcare professionals working in intensive care units (ICUs) were infected with COVID-19.^15^ Giwa et al. estimated that at least 10% of healthcare professionals in Italy would become infected with ­COVID-19 despite their use of PPE.^16^ In a case series analyzed by Wang et al., out of 138 consecutive patients who were hospitalized due to COVID-19 in Wuhan, China, during January 2020, 30% were healthcare professionals.^17^

In January 2020, the CDC released guidelines on the decontamination process for reusing N95 masks.^18^ A variety of procedures can be followed for reusing these masks, but none of the known methods completely remove the associated risks.^18^ The existing systematic reviews refer only to use of masks in relation to the COVID-19 pandemic.^14^ Concerning other types of PPE, such as gloves, glasses and face shields, the existing systematic reviews are not specific to COVID-19, and have included reference to several agents that cause respiratory infections.^6^ Furthermore, we did not find any systematic reviews on the use of PPE such as gloves, glasses and face shields, for protection against COVID-19.

## OBJECTIVES

The objective of the present study was to identify, systematically evaluate and summarize the available scientific evidence regarding the efficacy, safety, duration of use and reuse of personal protective equipment (masks, face shields and glasses) for healthcare professionals, for protection against infection by SARS-CoV-2.

## METHODS

### Study model

This study was a rapid systematic review. The research protocol was registered on the OPENSCIENCE Framework.

### Inclusion criteria

The search was performed in accordance with the Preferred Reporting Items for Systematic Reviews and Meta-Analyses (PRISMA) guidelines. Concerning the type of studies, given that only a limited number of studies have been published so far, the purpose of this review was to map the knowledge of the subject and identify the designs of these studies according to their level of evidence. There was no restriction on the origin, language or publication status of the study.

### Phenomena of interest

Use of PPE (masks, face shields and goggles) for prevention of COVID-19 transmission among healthcare professionals and disinfection of PPE constituted the phenomena of interest.

### Types of participants

Except for laboratory studies, the types of participants considered for this systematic review were healthcare professionals in a hospital environment or outpatient setting.

### Types of intervention

Studies that evaluated the effectiveness and efficacy of several types of PPE, such as different types of masks, duration of use, use of goggles and face shield protectors, separately or in combination, and other techniques that helped to prevent contamination by COVID-19 among healthcare professionals, were assessed.

### Types of outcome

#### Primary outcomes

The primary outcomes were interventions and disinfecting materials that were effective for preventing COVID-19 contamination.

#### Secondary outcomes

The secondary outcomes considered were the following:

- PPE durability
- User satisfaction
- Cost

The secondary outcomes were not considered as inclusion criteria for the studies.

### Search methods for selecting studies

The search strategy was elaborated starting from the following research question: “What is the degree of effectiveness and safety of personal protective equipment (masks, face shields and glasses) for the protection of healthcare professionals against infection by SARS-CoV-2, and how can this equipment be safely used and reused?”

The searches were elaborated using Health Science Descriptors and were translated into each of the databases selected: Cochrane Library (Wiley); Embase (Elsevier); BVS Portal; Medical Literature Analysis and Retrieval System Online (MEDLINE, PubMed); CINAHL; Web of Science; Scopus; and Opengrey (https://opengrey.eu). The following descriptors were used: severe acute respiratory syndrome coronavirus 2”[Supplementary Concept] OR “severe acute respiratory syndrome coronavirus 2”[All Fields] OR “sars cov 2”[All Fields] AND “Respiratory Protective Devices”[MeSH Terms] or “Masks”[MeSH Terms] AND “Face Shield”[MeSH Terms]

A manual search was also conducted in the reference lists of the primary and secondary studies identified in the electronic search. The search strategies developed and used for each electronic database were performed on November 30, 2020, and are presented in Table 1. There were no restrictions on languages or forms of publication.

**Table 1.** Search strategy according to the corresponding database

Database	Search Strategy
Cochrane Library	#1 (COVID 19) OR (COVID-19) OR (2019 nCoV) OR (nCoV) OR (Covid19) OR (SARS CoV) OR (SARSCov2 or ncov*) OR (SARSCov2) OR (2019 coronavirus*) OR (2019 corona virus*) OR (Coronavirus (COVID 19)) OR (2019 novel coronavirus disease) OR (COVID 19 pandemic) OR (COVID 19 virus infection) OR (coronavirus disease 19) OR (2019 novel coronavirus infection) OR (2019 nCoV infection) OR (coronavirus disease 2019) OR (2019 nCoV disease) OR (COVID 19 virus disease) #2 (Respiratory Protective Devices) OR (Device, Respiratory Protective) OR (Devices, Respiratory Protective) OR (Protective Device, Respiratory) OR (Protective Devices, Respiratory) OR (Respiratory Protective Device) OR (Respirators, Industrial) OR (Industrial Respirators) OR (Industrial Respirator) or (Respirator, Industrial) OR (Gas Masks) OR (Gas Mask) OR (Mask, Gas) OR (Masks, Gas) OR (Respirators, Air-Purifying) OR (Air-Purifying Respirator) OR (Air-Purifying Respirators) OR (Respirator, Air-Purifying) OR (Respirators, Air Purifying) OR Mask* #3 Face Shield* #4 #1 AND #2 AND #3
PubMed	#1 “COVID-19” [Supplementary Concept] OR (COVID 19) OR (COVID-19) OR (2019-nCoV) OR (nCoV) OR (Covid19) OR (SARS-CoV) OR (SARSCov2 or ncov*) OR (SARSCov2) OR (2019 coronavirus*) OR (2019 corona virus*) OR (Coronavirus (COVID-19)) OR (2019 novel coronavirus disease) OR (COVID-19 pandemic) OR (COVID-19 virus infection) OR (coronavirus disease-19) OR (2019 novel coronavirus infection) OR (2019-nCoV infection) OR (coronavirus disease 2019) OR (2019-nCoV disease) OR (COVID-19 virus disease) OR (COVID-19 virus infection) #2 “Respiratory Protective Devices”[Mesh] OR (Device, Respiratory Protective) OR (Devices, Respiratory Protective) OR (Protective Device, Respiratory) OR (Protective Devices, Respiratory) OR (Respiratory Protective Device) OR (Respirators, Industrial) OR (Industrial Respirators) OR (Industrial Respirator) OR (Respirator, Industrial) OR (Gas Masks) OR (Gas Mask) OR (Mask, Gas) OR (Masks, Gas) OR (Respirators, Air-Purifying) OR (Air-Purifying Respirator) OR (Air-Purifying Respirators) OR (Respirator, Air-Purifying) OR (Respirators, Air Purifying) OR “Masks”[Mesh] OR (Mask*) #3 Face Shield* #4 #1 AND #2 AND #3
EMBASE	#1 ‘covid 19’/exp OR (COVID 19) OR (COVID-19) OR (2019-nCoV) OR (nCoV) OR (Covid19) OR (SARS-CoV) OR (SARSCov2 or ncov*) OR (SARSCov2) OR (2019 coronavirus*) OR (2019 corona virus*) OR (Coronavirus (COVID-19)) OR (2019 novel coronavirus disease) OR (COVID-19 pandemic) OR (COVID-19 virus infection) OR (coronavirus disease-19) OR (2019 novel coronavirus infection) OR (2019-nCoV infection) OR (coronavirus disease 2019) OR (2019-nCoV disease) OR (COVID-19 virus disease) #2 ‘gas mask’/exp OR Gasmask OR (respiratory protective devices) OR ‘mask’/exp OR mask* #3 Face Shield* #4 #1 AND #2 AND #3
LILACS	#1 MH:”Infecções por Coronavirus” OR (Infecções por Coronavirus) OR (Infecciones por Coronavirus) OR (Coronavirus Infections) OR (COVID-19) OR (COVID 19) OR (Doença pelo Novo Coronavírus (2019-nCoV)) OR (Doença por Coronavírus 2019-nCoV) OR (Doença por Novo Coronavírus (2019-nCoV)) OR (Epidemia de Pneumonia por Coronavirus de Wuhan) OR (Epidemia de Pneumonia por Coronavírus de Wuhan) OR (Epidemia de Pneumonia por Coronavírus de Wuhan de 2019-2020) OR (Epidemia de Pneumonia por Coronavírus em Wuhan) OR (Epidemia de Pneumonia por Coronavírus em Wuhan de 2019-2020) OR (Epidemia de Pneumonia por Novo Coronavírus de 2019-2020) OR (Epidemia pelo Coronavírus de Wuhan) OR (Epidemia pelo Coronavírus em Wuhan) OR (Epidemia pelo Novo Coronavírus (2019-nCoV)) OR (Epidemia pelo Novo Coronavírus 2019) OR (Epidemia por 2019-nCoV) OR (Epidemia por Coronavírus de Wuhan) OR (Epidemia por Coronavírus em Wuhan) OR (Epidemia por Novo Coronavírus (2019-nCoV)) OR (Epidemia por Novo Coronavírus 2019) OR (Febre de Pneumonia por Coronavírus de Wuhan) OR (Infecção pelo Coronavírus 2019-nCoV) OR (Infecção pelo Coronavírus de Wuhan) OR (Infecção por Coronavirus 2019-nCoV) OR (Infecção por Coronavírus 2019-nCoV) OR (Infecção por Coronavírus de Wuhan) OR (Infecções por Coronavírus) OR (Pneumonia do Mercado de Frutos do Mar de Wuhan) OR (Pneumonia no Mercado de Frutos do Mar de Wuhan) OR (Pneumonia por Coronavírus de Wuhan) OR (Pneumonia por Novo Coronavírus de 2019-2020) OR (Surto de Coronavírus de Wuhan) OR (Surto de Pneumonia da China 2019-2020) OR (Surto de Pneumonia na China 2019-2020) OR (Surto pelo Coronavírus 2019-nCoV) OR (Surto pelo Coronavírus de Wuhan) OR (Surto pelo Coronavírus de Wuhan de 2019-2020) OR (Surto pelo Novo Coronavírus (2019-nCoV)) OR (Surto pelo Novo Coronavírus 2019) OR (Surto por 2019-nCoV) OR (Surto por Coronavírus 2019-nCoV) OR (Surto por Coronavírus de Wuhan) OR (Surto por Coronavírus de Wuhan de 2019-2020) OR (Surto por Novo Coronavírus (2019-nCoV)) OR (Surto por Novo Coronavírus 2019) OR (Síndrome Respiratória do Oriente Médio) OR (Síndrome Respiratória do Oriente Médio (MERS)) OR (Síndrome Respiratória do Oriente Médio (MERS-CoV)) OR (Síndrome Respiratória do Oriente Médio por Coronavírus) OR MH:C01.925.782.600.550.200$ #2 (Respiratory Protective Devices) OR (Device, Respiratory Protective) OR (Devices, Respiratory Protective) OR (Protective Device, Respiratory) OR (Protective Devices, Respiratory) OR (Respiratory Protective Device) OR (Respirators, Industrial) OR (Industrial Respirators) OR (Industrial Respirator) or (Respirator, Industrial) OR (Gas Masks) OR (Gas Mask) OR (Mask, Gas) OR (Masks, Gas) OR (Respirators, Air-Purifying) OR (Air-Purifying Respirator) OR (Air-Purifying Respirators) OR (Respirator, Air-Purifying) OR (Respirators, Air Purifying) OR Mask* OR (MASCARAS) #3 Face Shield$ #4 #1 AND #2 AND #3
SCOPUS	#1 (COVID 19) OR (2019 nCoV) OR (nCoV) OR (Covid19) OR (SARS CoV) OR (SARSCov2 or ncov*) OR (SARSCov2) OR (2019 coronavirus*) OR (2019 corona virus*) #2 (Respiratory Protective Devices) OR (Device Respiratory Protective) OR (Devices Respiratory Protective) OR (Protective Device* Respiratory) OR MASK* #3 Face Shield* #4 #1 AND #2 AND #3
Web of Science	#1 (COVID-19) OR (COVID 19) OR (COVID-19) OR (2019-nCoV) OR (nCoV) OR (Covid19) OR (SARS-CoV) OR (SARSCov2 or ncov*) OR (SARSCov2) OR (2019 coronavirus*) OR (2019 corona virus*) OR (Coronavirus (COVID-19)) OR (2019 novel coronavirus disease) OR (COVID-19 pandemic) OR (COVID-19 virus infection) OR (coronavirus disease-19) OR (2019 novel coronavirus infection) OR (2019-nCoV infection) OR (coronavirus disease 2019) OR (2019-nCoV disease) OR (COVID-19 virus disease) OR (COVID-19 virus infection) #2 (Respiratory Protective Devices) OR (Device, Respiratory Protective) OR (Devices, Respiratory Protective) OR (Protective Device, Respiratory) OR (Protective Devices, Respiratory) OR (Respiratory Protective Device) OR (Respirators, Industrial) OR (Industrial Respirators) OR (Industrial Respirator) or (Respirator, Industrial) OR (Gas Masks) OR (Gas Mask) OR (Mask, Gas) OR (Masks, Gas) OR (Respirators, Air-Purifying) OR (Air-Purifying Respirator) OR (Air-Purifying Respirators) OR (Respirator, Air-Purifying) OR (Respirators, Air Purifying) OR Mask* #3 Face Shield* #4 #1 AND #2 AND #3
CINAHL	#1 (COVID-19) OR (COVID 19) OR (COVID-19) OR (2019-nCoV) OR (nCoV) OR (Covid19) OR (SARS-CoV) OR (SARSCov2 or ncov*) OR (SARSCov2) OR (2019 coronavirus*) OR (2019 corona virus*) OR (Coronavirus (COVID-19)) OR (2019 novel coronavirus disease) OR (COVID-19 pandemic) OR (COVID-19 virus infection) OR (coronavirus disease-19) OR (2019 novel coronavirus infection) OR (2019-nCoV infection) OR (coronavirus disease 2019) OR (2019-nCoV disease) OR (COVID-19 virus disease) OR (COVID-19 virus infection) #2 (Respiratory Protective Devices) OR (Device, Respiratory Protective) OR (Devices, Respiratory Protective) OR (Protective Device, Respiratory) OR (Protective Devices, Respiratory) OR (Respiratory Protective Device) OR (Respirators, Industrial) OR (Industrial Respirators) OR (Industrial Respirator) or (Respirator, Industrial) OR (Gas Masks) OR (Gas Mask) OR (Mask, Gas) OR (Masks, Gas) OR (Respirators, Air-Purifying) OR (Air-Purifying Respirator) OR (Air-Purifying Respirators) OR (Respirator, Air-Purifying) OR (Respirators, Air Purifying) OR Mask* #3 Face Shield* #4 #1 AND #2 AND #3

### Selection of studies and data extraction

Identification of eligible studies followed a two-stage process accomplished by two independent reviewers. Any disagreement was resolved by a third reviewer. In the first stage, after exclusion of duplications, the titles and abstracts of the references identified through the search strategy were evaluated, and the potentially eligible studies were preselected. In the second stage, a full-text evaluation on the studies preselected was carried out to confirm their eligibility. The selection process was performed through the Rayyan platform (https://rayyan.qcri.org).^19^ The details of the ten studies that in the end were selected for evaluation are shown in Table 2.^7^^,^^8^^,^^20^^,^^21^^,^^22^^,^^23^^,^^24^^,^^25^^,^^26^^,^^27^

**Table 2.** Analysis of the articles included in the study

Study and country	Study design	Sample	Material analyzed	Results and conclusion
Armijo et al.^25^ United States	Laboratory.	The experiments were repeated five times on the face shield (headband, headpiece, facial shield) and organism (*Escherichia coli*, *Staphylococcus aureus*), thus making a total of 30 experiments, not including positive and negative controls.	In total, 112 face shields (the solid headband and the chin protector part of the face shield) were printed in 3D on the FDM platform because they were more accessible and easier to use for non-industrial applications. Diluted bleach solution was used for decontamination. *Escherichia coli* and *Staphylococcus aureus* were selected as Gram-negative and Gram-positive model organisms.	Face shields were useful and inexpensive. The efficacy of the decontamination protocol against *Escherichia coli* was greater than that of *Staphylococcus aureus*. *E. coli* was observed on facial protection, *Staphylococcus aureus* was detected on facial protection and on the chin. No organisms were recovered from the head bands. The decontamination protocol was highly effective against *Escherichia coli* and *Staphylococcus aureus*, achieving a reduction ≥ 4 log 10 (99.99%) in colony counts for each repeat. Face shields formed a barrier against soiling of N95 face masks and were more effective for eye protection from respiratory droplets than standard eye shields. Implementation of decontamination protocols successfully allowed face shield and N95 mask reuse, thus enabling a higher level of protection for anesthesiology providers at the onset of the COVID-19 pandemic.
Arumuru et al.^24^ India	Laboratory, using a standard mannequin in a controlled environment.	Homemade cotton mask, surgical mask, N95 mask and a nostril of a mannequin.	Homemade masks, N95 masks and surgical masks, and a 30,000 Reynolds pulsed jet sneeze simulator. Trace particles were introduced into the stream to capture the emulated turbulent jet formed due to a sneeze. Compressed air and a solenoid valve were also used. A laser camera and lighting were set up.	A homemade three-layer mask was suitable for preventing penetration of fine-sized particles, but in a sneeze, these can travel up to 45.7 cm. With a surgical mask, the sneeze particles can travel up to approximately 76.2 cm and with a surgical mask plus a face shield the spread of the particles become greater, by traveling 12.2 cm. An N95 mask blocks sneezing in the forward direction; however, leakage from the sides and top spreads the sneeze backwards over a distance of up to approximately 60.9 cm. None of the measures adopted, such as homemade two- and three-layer masks, standard three-layer surgical masks and face shields effectively blocked the escape of particles ejected during sneezing. Protective measures effectively reduced leakage and diminished the sneeze range to between 30 and 90 cm.
Chaturvedi et al.^7^ India	Cross-sectional study analyzing the characteristics of 3D printed face shields.	227 healthcare professionals.	Face shield produced by 3D printer.	Orthopedic surgeons reported that the face shield was useful during screening tasks, in which the interactions with patients involved wound care, immobilization and application of traction. In the wards and in the ICU, all groups of healthcare professionals found that face shields with soft PVC film were effective during airway management and other aerosol-generating procedures, as they could insert the PVC film visor into the PPE gown to provide complete closure of the facial region, although with an opening at the top for ventilation. Development of face shields with participation by healthcare professionals increased their acceptability and effectiveness. Use of face shields was effective in screening and treatment situations, with the ability to vary the configuration of the device. Cost-efficacy, ergonomics, reuse and acceptance were evaluated among orthopedic surgeons and emergency medicine personnel and positive feedback was obtained in relation to all variables considered.
Chow et al.^23^ China	Cross-sectional study in a real environment.	Five patients without clinical evidence of COVID-19 underwent tracheostomy. Two horizontal anesthetic screens and a transparent sterile plastic sheet (plastic curtain) over a tracheostomy operative field were used.	Presence or absence of droplet contamination on the five plastic sheets used (plastic curtain) during the procedures and on the surgeon’s face shield and instrumentation.	All five sheets were contaminated with droplets of 0.2 to 2.8 mm. Droplet contamination was most severe on the central surface at 91.5% (range: 86.7%-100.0%) followed by the left and right-side surfaces at 5.2% (6.7%-10.0%) and 3.3% (6.7%-10.0%), respectively. No droplet contamination was observed on the face shield. The droplet contamination count was greater in the upper central half of the plastic sheet that covered the surgical site in the lower part of the neck. Use of two horizontal anesthetic screens and a sterile plastic sheet over a tracheostomy operative field can effectively prevent droplet contamination, thus eliminating the need for a face shield with adequate eye protection and respirator. No droplet contamination was observed on the surgeon’s face shield or on the instruments, thus showing that the plastic sheets (plastic curtain) were effective in preventing droplet and aerosol spillage.
Fischer et al.^22^ United States	Research letter citing a cross-sectional laboratory study.	N95 mask.	Ultraviolet light (260-285 nm) Dry heat at 70 °C. 70% alcohol. VHP.	The decontamination method against SARS-CoV-2 on N95 masks considered the time needed to reduce the virus viability within 1000 minutes. With ethanol, 99.56% was eliminated; with dry heat (70 °C), 93.89%; with UV light (260-285 nm), 91.84%; and with vaporized VHP, 99.36%. All the substances analyzed decontaminated the N95 masks. Decontamination by means of vaporization with hydrogen peroxide or ultraviolet light allowed N95 masks to be reused three times, while doing this with dry heat at 70 °C allowed it twice. Decontamination with 70% alcohol reduced the integrity of N95 masks and was not recommended.
Noguera et al.^20^ Brazil	Cross-sectional study with evaluation questionnaire applied to users of the face shields that were developed.	3D printing face shield: the total number evaluated was not reported.	Face shield after chemical disinfection: [70% ethanol, H_2_O_2_-quaternary ammonium salt mixture, 0.1% sodium hypochlorite or water (negative control)] with different thicknesses and materials were tested: 0.5 mm and 0 mm polyethylene glycol, 75 mm, 0.75 mm polycarbonate, 0.5 mm PET and 0.5 mm glycol modified PETG. Headbands for face shield after chemical disinfection and autoclaving (121 °C for 15 minutes): Different materials (Tritan HT, PLA EasyFill, ASA WP, ABS PT and PETG XT) and different layer thicknesses (0.15 mm, 0.30 mm, 0.60 mm) were used. 3D printing questionnaire: Online about the comfort, visual integrity and viability of the 3D face shield.	3,343 hospitalized COVID-19 patients, 2,778 trained health workers and 30,000 face shields were used. Face shield visual integrity after chemical disinfection None of the materials of the face shields or the layer thickness were damaged after a maximum of 40 disinfections with 70% ethanol, a mixture of quaternary ammonium salts and 0.1% sodium hypochlorite. To reduce the potential damage from steam, it is recommended to wait 3-5 minutes after each disinfection, given that at one minute after disinfection with 70% alcohol, vapors can cause eye redness. Headbands for face shield after chemical disinfection and autoclaving After chemical disinfection 30 times, none of the headbands had changes to their visible physical structure, as occurred with the mask visors. After decontamination in an autoclave, the PETG XT and TRITAN HT supports were found to have suffered considerable damage. There were reductions in size and material conditioning, and some cracks appeared, through the effect of the temperature and pressure of the autoclave; which led to a reduction in resistance through triggering of microfiber buckling. 3D printing questionnaire about face shields In the questionnaire, most answers were very good, with regard to mobility, visual integrity, mask removal and disinfection. All projects were considered adequate, with no major differences between them. The GRU and INSPER projects received higher marks from users.
Ong et al.^8^ Singapore	Cross-sectional study using a standardized technique with pre-moistened sterile smears.	Eyeglass protection, N95 respirators and shoe surfaces of 30 healthcare professionals who cared for 15 patients.	Sampling study on PPE used for one day by healthcare professionals who were taking care of confirmed COVID-19 patients over the previous 48 hours. All patients were in isolation rooms for airborne infections with 12 air changes per hour.	All 90 samples from 30 healthcare professionals (doctors, nurses and cleaning professionals) were negative. The average time spent in the patient’s room in general was 6 minutes (range: 5-10): 8 minutes for doctors, 7 minutes for nurses and 3 minutes for cleaning professionals. The activities ranged from casual contact (e.g. medication administration or cleaning) to closer contact (e.g. physical examination or collection of respiratory samples). Prolonged use of N95 masks and eyeglass protection with strict adherence to environmental and hand hygiene when handling patients with SARS-CoV-2 may be a safe option. These results may not be generalizable to other room configurations.
Saini et al.^26^ India	Laboratory.	Personal protective clothing, N95 masks and face shields obtained in a biosafety level 3 laboratory and in a hospital.	Biological indicators and culturing conditions Biological indicator strips with *B. stearothermophilus* were used as the gold standard to confirm the integrity of the sterilization process. Recombinant laboratory strains of *E. coli* and *M. smegmatis* were incorporated into the study to assess their suitability as a biological indicator for disinfecting personal protective equipment. Heat and alcohol treatment 70 °C and 80 °C for 5 and 10 minutes each, 75% and 85% ethanol for 0.5 and 1 min each and propan-2-ol (75% and 85% for 0.5 and 1 minute each. Disinfection using VHP Run time of around 10 minutes with 200 ml hydrogen peroxide solution for a 1000 cubic foot room.	Biological indicators for disinfection of PPE for SARS-CoV-2 *E. coli* was used as an indicator: it completely lost its viability at 70 °C and 85 °C. *Mycobacterium smegmatis* was more resistant to heat. *Escherichia coli* exhibited a low level of ethanol resistance, while *Mycobacterium smegmatis* was not viable. Use of propan-2-ol allowed viability of *Escherichia coli* and *Mycobacterium smegmatis*. Gold-standard *Bacillus stearothermophilus* spores exposed to aggressive treatments (heat 90 °C/30 minutes or alcohol 85%/1 minute) showed rebirth and growth. Only the *Bacillus stearothermophilus* standard remained viable under all conditions known to inactivate the SARS-CoV-2 virus, thus indicating its versatility as an ideal substitute or biological indicator for developing disinfection protocols for COVID-19. Disinfection using vaporized hydrogen peroxide (VHP) *Escherichia coli* was sterilized and there was a reduction greater than 7 log_10_ in *Mycobacterium smegmatis*. *Bacillus stearothermophilus* spores did not revive with VHP. A single VHP cycle (7%-8% hydrogen peroxide) was able to disinfect PPE in less than 10 min. Repetition of the procedure did not result in any physical break, deformity or other considerable change to the overalls and N95 masks.
Sapoval et al.^21^ France	Cross-sectional study.	38 radiologists (21 attending physicians, 6 fellows and 11 residents) in 31 consecutive interventions, such as central venous access, percutaneous peripheral angioplasty, percutaneous urinary intervention, arterial embolization due to acute bleeding, radiofrequency ablation of lung tumor, transjugular liver biopsy and sampling of the adrenal vein.	Face shields consisting of a standard transparent polymerizable vinyl chloride sheet were built on a 3D printer. The 3D printed face shields were evaluated in 31 interventional procedures. The average duration of the interventions was 59 ± 58 (SD) minutes (range: 15-240 minutes). Each face shield was used 2 ± 1.7 (SD) times (range: 1-8 times)*.*	In total, the average duration of the interventions was 59 ± 58 (SD) minutes (range 15-240 minutes); each face shield was used 2 ± 0.8 (SD) times (range 1-8 times). The average rating for the ability to perform the intervention assigned as usual was 1.7 ± 0.8 (SD) (range: 1- 4). The average visual tolerance rating was 1.6 ± 0.7 (SD) (range: 1-4). The average tolerability rating was 1.4 ± 0.7 (SD) (range: 1-3). Visual tolerance was satisfactory and no discomfort was observed, even during lengthy interventions. The study showed that 3D printed face shields were well accepted in several interventions in interventional radiology.
Smith et al.^27^ United States	Simulation study in an ICU environment.	A simulated patient in an ICU, with infection due to severe acute respiratory syndrome coronavirus-2 that involved endotracheal intubation, was used. A laryngoscopist, a nurse and a respiratory therapist assisted in laryngoscopy. Three different methods of intubation were used. Fluorescent marker was sprayed by means of an atomizer during the procedure. The three techniques included only PPE, a polycarbonate intubation box or a flexible coronavirus enclosure. Black light was used to evaluate the laryngoscopist and the respiratory therapist.	Contamination of the professional and personal protective equipment (gloves, apron, shoes and face shield) The PPE consisted of two masks (an N95 that was covered with a conventional surgical mask to protect the N95), a face shield, a cap, a long-sleeved waterproof plastic apron and gloves.	One person can install the coronavirus flexible casing in about two minutes. The intubation box can be unfolded in about two minutes, but it needs two people to position it properly. Use of PPE alone seriously contaminated the laryngoscopist and respiratory therapist. With the use of the intubation box, contamination of the laryngoscopist occurred only on the gloves, while the apron and face shield were not contaminated. The respiratory therapist showed great contamination on the gloves, the apron and the neck and face shield. The laryngoscopist reported that the arm holes restricted movement a little, without compromising intubation. With the coronavirus flexible closure system, the laryngoscopists and respiratory therapists were more protected. Only the gloves of both were contaminated. With all the three types, neither the nurse nor the surroundings were contaminated. The coronavirus flexible enclosure contained the fluorescent marker more effectively during endotracheal intubation than did personal protective equipment alone or the intubation box, based on the exposure of the laryngoscopist and respiratory support therapist.

ICU = intensive care unit; FDM = fused deposition modeling; VHP = vaporized hydrogen peroxide; PET = polyethylene terephthalate; PETG = polyethylene terephthalate glycol; SD = standard deviation; PPE = personal protective equipment; PVC = polyvinyl chloride; SARS-CoV-2 = severe acute respiratory syndrome coronavirus 2; UV = ultraviolet; HPV = human papillomavirus.

### Evaluation of methodological quality

The critical appraisal tool of the Joanna Briggs Institute was applied to all eligible studies in order to evaluate the methodological quality of the studies.

## RESULTS

### Studies selected

**Figure 1.**
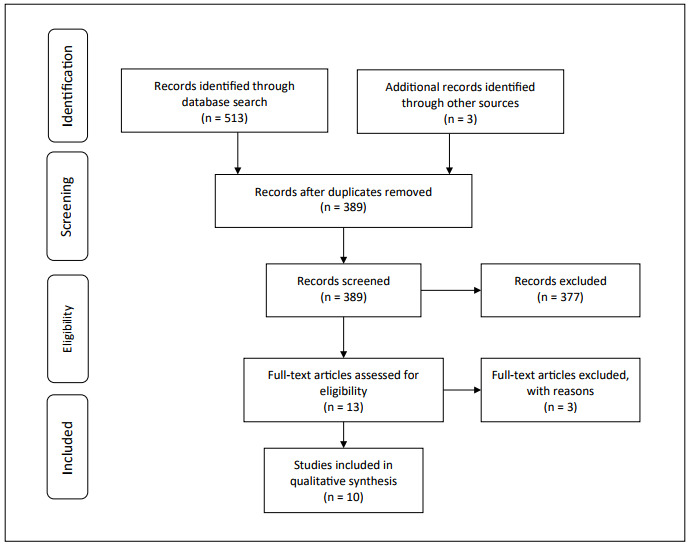
PRISMA flow diagram for study selection.

The systematic review yielded 513 papers and a further three papers were identified through manual searches. After removing duplicates, we obtained 389 articles. After the titles and abstracts had been read by two independent evaluators through the Rayyan online platform, 13 articles were included for reading the full text. After the full texts had been read, another three studies were excluded. The PRISMA flowchart is shown in Figure 1. Thus, ten studies were included for analysis. Since these studies refer to COVID-19, all of them were from the year 2020: two were published in June, two in July and one in each of the following months: March, August, September, October, November and December.

### Characteristics of studies included

The ten studies included all related to COVID-19 and were from the year 2020. Two of them were published in June, two in July and one in each of the following months: March, August, September, October, November and December. One article was produced in Brazil, three in the United States, one in France, one in China, three in India and one in Singapore.

Six studies were cross-sectional (Ong, Chaturvedi, Noguera, Sapoval, Fischer and Chow),^7^^,^^8^^,^^20^^,^^21^^,^^22^^,^^23^ three studies had laboratory designs (Arumuru, Armijo and Saini)^24^^,^^25^^,^^26^ and one study (Smith) was carried out on a simulation mannequin in an intensive care setting.^27^

Ong et al.^8^ sampled the PPE used by healthcare professionals who cared for patients with COVID-19. Chaturvedi et al.^7^ surveyed the opinions of 227 orthopedic surgeons and emergency medicine professionals who made use of 3D printed face shields. Noguera et al.^20^ tested chemical disinfectants and autoclaving on 3D printed face shields, and also investigated the comfort, viability and visual integrity of the shields through a questionnaire that was applied to the healthcare professionals who were using them.

Sapoval et al.^21^ conducted a study among 38 interventionist radiologists to assess the visual comfort of 3D printed face shields and these professionals’ tolerance of them and ability to perform interventions normally while using them. Fischer et al.^22^ analyzed decontamination of N95 masks and the possibility of their reuse. Chow et al.^23^ used plastic curtains draped across the patients during tracheostomy and head and neck surgery, with the aim of minimizing the contamination of healthcare professionals from droplets and aerosols emanating from the patients, during the procedures. These authors analyzed the plastic sheets of the curtain and the face shields of the professionals to identify contamination.

Arumuru et al.^24^ used a standard laboratory mannequin that simulated sneezing, in order to assess the effectiveness of masks for blocking the particles ejected during sneezing. Armijo et al.^25^ analyzed protocols for decontaminating 112 face shields, in a laboratory. Saini et al.^26^ evaluated the disinfection process for personal protective clothing, N95 masks and face shields, in a laboratory.

Smith et al.^27^ conducted a simulation study in an ICU, among three healthcare professionals, to assess the risk of contamination of professionals and the use of PPE with three different protection strategies.

### PPE analyzed

#### N95, surgical and cotton masks

Ong et al.^8^ evaluated the front of the goggles and the front of the N95 masks of professionals who cared for 15 patients with COVID-19 in isolation rooms. These were each used during one day of activity and no contamination was found on these materials, thus confirming that prolonged use of N95 masks and goggles with adequate environmental and hand hygiene is a safe option. Arumuru et al.^24^ evaluated the effectiveness of homemade cotton masks with three layers, N95 masks, standard three-layer surgical masks and face shields, using a sneeze simulator. They concluded that none of these measures effectively blocked the escape of particles ejected during sneezing. Protective measures effectively reduced leakage and shortened the sneeze range to between 30 and 90 cm. These authors stated that, without a mask, particles from a common sneeze can be projected for approximately 760 cm (25 ft) in almost 22 seconds. The N95 masks completely prevented the particles from leaking forwards, but leakage could still occur sideways and could move up to 60 cm backwards.

### Face shields

Three studies on 3D-printed face shields and one study on N95 masks met our inclusion criteria for this investigation. Face shields produced through 3D printing were presented as an easy-to-implement economical alternative that would promote safety for users against infection by COVID-19 aerosols. One important point presented in these articles related to reducing the contamination and dirt of N95 masks, so as to favor longer-duration use and provide additional protection.

Chaturvedi et al.^7^ evaluated 3D printed face shields in terms of cost-effectiveness, ergonomics, reuse and acceptance by orthopedic surgeons and emergency medical personnel. They reported that there was positive feedback regarding all variables.

On the other hand, Noguera et al.^20^ evaluated low-cost 3D-printed face shields for use by healthcare professionals during their shifts for treating patients with COVID-19. The disinfection protocols used on these shields were tested, and the comfort, visual integrity and viability of the protectors were evaluated by these professionals. These shields were found to be well accepted by the professionals. Chemical disinfection with 70% ethanol, 0.1% sodium hypochlorite and a mixture of quaternary ammonium and H_2_O_2_ was effective and did not cause changes to the materials of the face shields. Nonetheless, autoclaving has been shown to cause physical changes to face shields and should not be used.

The study carried out among interventional radiologists by Sapoval et al.^21^ aimed to clinically evaluate face shields printed on a 3D printer with regard to protection against droplets from ­SARS-CoV-2, through 31 interventional procedures. Satisfactory results regarding visual comfort and tolerance were obtained.

Armijo et al.^25^ aimed to meet the demands of anesthesiologists regarding the safety of masks to protect against COVID-19 infection. They also sought to minimize the contamination and dirt of N95 masks, so as to favor their reuse through an ultraviolet radiation sterilization protocol. Face shields were found to be useful, with low cost, and the decontamination protocol was highly effective against *Escherichia coli* and *Staphylococcus aureus* in the tests performed.

### Protective goggles

No specific studies on goggles that met the inclusion criteria were identified.

### Flexible enclosure with plastic cover or polycarbonate intubation box

A flexible enclosure with a plastic cover or polycarbonate intubation box was used in a study by Smith et al.,^27^ with testing during an endotracheal intubation procedure in an intensive care unit. A simulated patient with COVID-19 was attended by a laryngoscopist, a respiratory therapist who assisted in the intubation and a nurse. Both the PPE used (gloves, apron, shoes and face shields) and the healthcare professionals themselves were evaluated for contamination, by means of fluorescent markers that were sprayed out by an atomizer throughout the procedure. The flexible enclosure for coronavirus was found to contain the fluorescent marker more effectively during endotracheal intubation than PPE alone or the intubation box, based on the exposure of the laryngoscopist and respiratory support therapist.

### Plastic curtains

Plastic curtains were tested in a study by Chow et al.^23^ during five surgical procedures that involved tracheostomy and head and neck surgery. Droplet contamination was observed on all the plastic sheets that made up the curtain, such that this contamination was highest on the central surface, then on the left-side surface and then on the right. No contamination was seen on the face shields. Thus, this device can minimize transmission of the virus to healthcare professionals.

### PPE disinfection and cleaning

Noguera et al.^20^ aimed to evaluate the use of 3D printed face shields for comfort, durability, visual integrity and viability, and ways of disinfecting them. They observed that simple forms of disinfection, used conventionally, were safe and effective, except for autoclaving, which could cause significant damage to some materials used in making face shields.

Armijo et al.^25^ aimed to provide additional protection for healthcare professionals, at low cost, that would reduce the potential contamination of N95 masks, thereby favoring longer duration of use with easy decontamination. They concluded that the use of N95 with face shields was useful and that the protocol for decontamination with a diluted bleach solution allowed penetration into any of the pores generated in the 3D printing process of the face shield, such that the decontamination process was highly effective.

Saini et al.^26^ aimed to evaluate biological indicators as positive sterilization controls and to develop a method using vaporized hydrogen peroxide. They demonstrated that there was no impairment of the materials and no alteration of comfort perceived by the user.

Fischer et al.^22^ aimed to compare methods for decontamination of N95 masks using ultraviolet light (260-285 nm), dry heat at 70 °C, 70% alcohol and vaporization with hydrogen peroxide. Vaporization with hydrogen peroxide showed the best results concerning SARS-CoV-2 and preservation of the integrity of N95 masks. Ultraviolet light eliminated SARS-CoV-2 more slowly and preserved the function of the N95 mask almost as well as vaporization with hydrogen peroxide. Both of these techniques allowed N95 masks to be reused three times.^22^ Dry heat at 70 °C eliminated the virus with a speed similar to ultraviolet light, and was likely to maintain acceptable adjustment values for one or two decontaminations.^22^ Decontamination with 70% alcohol reduced the integrity of N95 masks and was not recommended.^22^

### Satisfaction of healthcare professionals in relation to PPE

Chaturvedi et al.^7^ aimed to analyze the effectiveness of face shields made in a 3D printer that were used by orthopedists and emergency medical professionals. These professionals considered them to be satisfactory for use.

Sapoval et al.^21^ aimed to evaluate the risk factors for COVID-19 among interventional anesthesiologists who used 3D-printed face shields during routine procedures in hospitals. It was concluded that 3D-printed face shields were well-accepted by anesthesiologists and fulfilled the proposed objectives.

## DISCUSSION

The main contribution of this review was to synthesize the best available evidence about the use, reuse and disinfection of PPE as a non-pharmacological intervention, used in association with other measures for preventing contamination by ­COVID-19, such as cough etiquette (a common cough can travel at least 1.5-3 meters)^24^ and hand hygiene, for healthcare professionals.

The main point for management of COVID-19 infection is to prevent in-hospital infection among healthcare professionals.^28^ Coronavirus can spread through aerosols, droplets and contact contamination.^7^ A droplet of 10 μm in diameter may persist suspended in the air for about 50 seconds when sneezing.^24^ Since the half-life of the virus is approximately 6-7 hours, it is unlikely that there will be a viable amount of virus in the mask for three full days.^29^ van Doremalen et al.^29^ analyzed the survival of SARS-CoV-2 with 40% humidity at temperatures of 70-73 °F (21-22 °C), and concluded that it could survive as follows:

- Up to 3 hours after aerosolization, with an average half-life of 1.1 to 1.2 hours.
- Up to 4 hours on copper, with an average half-life of 1.1 to 1.2 hours.
- Up to 24 hours on cardboard, with an average half-life of approximately 3.5 hours.
- Up to 2 days on stainless steel, with an average half-life of 5.6 hours.
- Up to 3 days on plastic, with an average half-life of 6.8 hours.

Face shields have been classified as a form of PPE that protects the facial area and related mucous membranes (ear, nose and mouth) from splashes of body fluids.^7^^,^^30^ Among the four types of PPE for the facial region, namely, face shields, face shields with N95 respirator, surgical masks with eye protector and safety glasses with N95 respirator, face shields with N95 respirator are the most effective.^7^ The association of a face shield with N95 respirator and a flexible enclosure with a plastic cover or polycarbonate intubation box, as demonstrated by Smith et al.,^27^ along with the plastic curtain that was used in the study by Chow et al.,^27^ may be of special importance for protection of healthcare professionals during procedures such as orotracheal intubation and procedures involving tracheostomy.

Use of face shields can substantially reduce healthcare professionals’ short-term exposure to larger particles of infectious aerosols and can reduce the contamination of their respirators.^11^^,^^31^^,^^32^ They are less effective against smaller particles, which can remain in the air for long periods and easily flow around a face mask, for inhalation.^11^ Thus, face shields can provide a useful complement to respiratory protection for workers who care for patients with respiratory infections but cannot be used as a substitute for respiratory protection.^11^^,^^32^ Although face shields are bulkier than goggles or safety glasses, they offer the advantage of protecting the entire face from contamination.^11^^,^^30^ Some professionals may also feel more comfortable with face shields or may find that they fit better than glasses or respirators.^11^^,^^30^

During the pandemic, cheaper solutions have emerged that can be developed locally, such as face shields, masks, etc.^7^ These solutions are being widely propagated in urban areas with large-scale 3D printing facilities.^7^ In the situation of lockdowns to which many regions have been subjected, domestic production becomes crucial for obtaining the best functional results regarding production of PPE.^7^ Production of face shields is simple: they consist of three parts, i.e. the frame, an elastic cord for attachment and a transparent plastic visor.^7^^,^^32^ When produced using a 3D printer, the total cost of one face shield is approximately one dollar, which makes it an economical device, considering its reusability.^7^^,^^32^

Before disinfecting face shields, they need to be disassembled.^7^ Polyvinyl chloride (PVC) film visors should be discarded after use and replaced for the next cycle of use.^7^ The disinfection procedures to be followed are as per the recommendations of the CDC, using standard disinfection solutions such as isopropyl alcohol or sodium hypochlorite, and subsequently performing proper hand hygiene.^7^ It is advisable to discard the paper clips or band after one cycle of use. Nonetheless, in situations that require reuse, they can be disinfected using a hospital disinfectant solution that has been registered with the Environmental Protection Agency (EPA).^7^

This information is decisive when thinking about mask reuse and the cost of masks. In March 2020, the U.S. Department of Health and Human Services announced that its national inventory strategy, i.e. the emergency stock of medicines and medical supplies, contained approximately 42 million masks, totaling both surgical and N95 masks.^14^^,^^33^ This is equivalent to 1% of the estimated amount needed by United States healthcare professionals in a pandemic scenario (42 million stored compared with the estimated 3.5 billion needed).^14^^,^^33^ In addition, use of face shields reduces mask contamination, when they are used together, thus extending their useful life.^25^

Although face shields do not offer absolute protection against contamination, they significantly decrease the chances of contracting the coronavirus.^7^ One of the main problems with face shields and PPE hoods is the fogging of the visor, which impairs users’ abilities during procedures and surgeries.^7^ Discomfort due to lack of adequate ventilation is also a considerable concern.^7^

Li et al. analyzed masks after disinfection with 75% alcohol or soap and water at 60 °C and noted that the structure of the medical masks was damaged after treatment with these substances.^31^ Use of water and soap or alcohol significantly reduces the filtering efficiency of N95 masks (54% and 67%, respectively).^34^ Treating these masks with gamma radiation is also not recommended.^34^ Autoclaving is not indicated for disinfection of face shields printed on 3D printers since it can cause significant damage to some materials used in their manufacture.^20^ Chemical disinfection with 70% ethanol, 0.1% sodium hypochlorite and a mixture of quaternary ammonium and H_2_O_2_ is indicated.^20^ Decontamination with diluted bleach solution is also an option.^25^ Moreover, diluted bleach solution is useful for disinfection from some bacteria, such as *Staphylococcus aureus*.^25^

SARS-CoV-2 has been found to be highly stable at 4 °C but sensitive to heat.^35^ According to the inventor of the N95 mask material, Dr. Peter Tsai, this material can be heated for 60 minutes, steamed at 125 °C for five minutes or boiled for five minutes, and then air-dried.^34^ Most viruses are killed in less than two minutes when the water temperature reaches 70 °C (158 °F).^34^^,^^36^ The rubber band must not be immersed in boiling water.^34^ Through these methods, 92.4% and 91.7%-98.5% of mask filtration efficiency is retained and the criteria required by the Food and Drug Administration (FDA) and the CDC are met.^34^ Nevertheless, if these methods are performed for more than five minutes, the efficiency of the filtration will drop further.^34^

Use of hydrogen peroxide was helpful in disinfecting PPE without damaging its material.^22^^,^^26^ Decontamination through vaporization with hydrogen peroxide showed the best results concerning the speed of inactivation of SARS-CoV-2 and preservation of the integrity of N95 masks.^22^ In the sequence, ultraviolet light eliminated SARS-CoV-2 more slowly and preserved the function of the N95 masks almost as well as vaporization with hydrogen peroxide, thus allowing the masks to be reused up to three times.^22^ Although dry heat at 70 °C eliminate the virus at a speed similar to that of ultraviolet light, it damages the masks and thus only allows reuse twice.^22^ Seventy percent alcohol impairs the integrity of N95 masks, and decontamination with this substance is not indicated.^22^^,^^37^

The ideal would be to use an N95 mask for one day and only use it again on the fifth day, which would therefore require at least four masks.^34^ All copies of SARS-CoV-2 on the mask will be dead within three days even if no decontamination is performed.^29^^,^^34^

Although quaternary ammonium or bleach can also be used to disinfect gloves,^10^ there is no evidence that they have any action on COVID-19. It should also be noted that there is little evidence that using two gloves on each hand as part of full-body PPE can reduce the risk of contamination and reduce the viral load on the hands without constant change of the gloves.^10^

None of the articles identified showed the ideal length of time for using the masks, or how to safely store them for reuse. However, the need to follow the recommendations of the company responsible for the product and those of the hospital’s infection control commission were indicated.^22^

### Implications for practice

The implications for practice of this review are that combined use of a face shield with a N95 mask among healthcare professionals may increase these professionals’ protection. This would enable them to have a lower rate of infection and would thus reduce the pressure on the healthcare system.

Emphasis should also be given to the possible disinfection of these materials with 70% ethanol, 0.1% sodium hypochlorite, bleaches and a mixture of quaternary ammonium with H_2_O_2_. Disinfection, with the possibility of reuse, reduces the demand for these PPE materials, which may reduce the cost to institutions.

Disinfection of N95 masks by means of vaporization using hydrogen peroxide or ultraviolet light made it possible to reuse these masks three times, while use of dry heat at 70 °C allowed reuse twice. Decontamination with 70% alcohol would reduce the filtration efficiency of N95 masks and is not recommended. The main findings from the systematic review are summarized in Table 2.

### Research implications

There is a need to assess the durability of PPE and how to store it properly after use and disinfection. It is also necessary to evaluate disposable gloves and aprons, in order to reduce the demand for these items, given the large number of exchanges necessary during the work periods of healthcare professionals and their increasing market price, which is increasing the costs of professionals, institutions and governments.

We identified that there is a need for randomized controlled studies and observational studies with adequate designs, in order to better identify the risk factors, effectiveness, safety and cost of preventive measures for healthcare professionals who are faced with the challenge of COVID-19.

## CONCLUSION

The studies identified so far provide low levels of evidence but consistently demonstrate that N95 masks, surgical masks and face shields, both those industrially manufactured and those produced through lower-cost 3D printers, are meaningful devices that act as a barrier to droplets and enable protection for healthcare professionals against COVID-19 infection. However, additional care regarding the length of time of use, disinfection and reuse is needed, along with hand hygiene and care regarding placement and removal of these devices. Combined use of a face shield and a N95 mask proved to be superior to separate use of each device or associations between face shields and surgical or cloth masks.

Auxiliary devices, such as flexible enclosures for coronavirus and plastic curtains, may be an additional alternative for protecting professionals who are directly involved in procedures with a higher risk of contamination, such as orotracheal intubation and tracheostomy. Some products are useful for disinfecting PPE, such as 70% ethanol, 0.1% sodium hypochlorite and a mixture of quaternary ammonium and hydrogen peroxide. Ultraviolet light and dry heat at 70 °C can be used to decontaminate N95 masks, while it needs to be borne in mind that dry heat at 70 °C reduces the integrity of N95 masks more dramatically.
